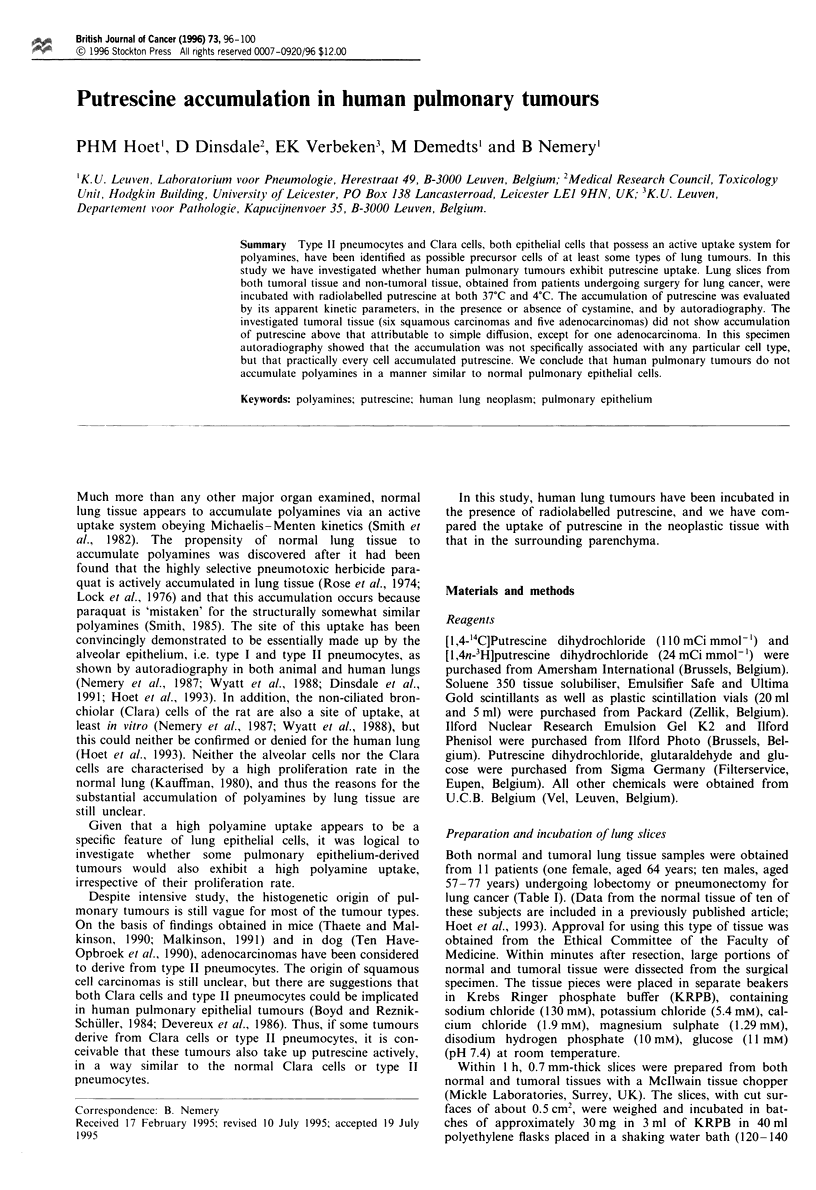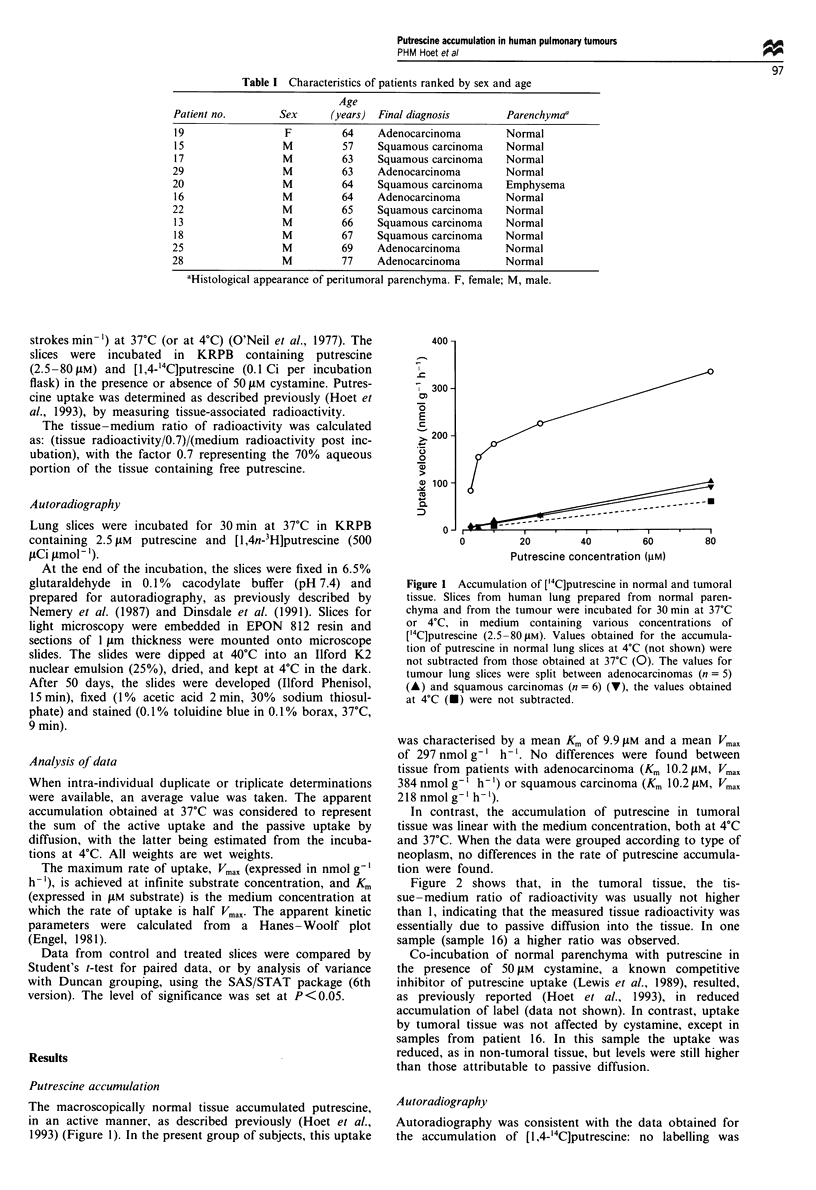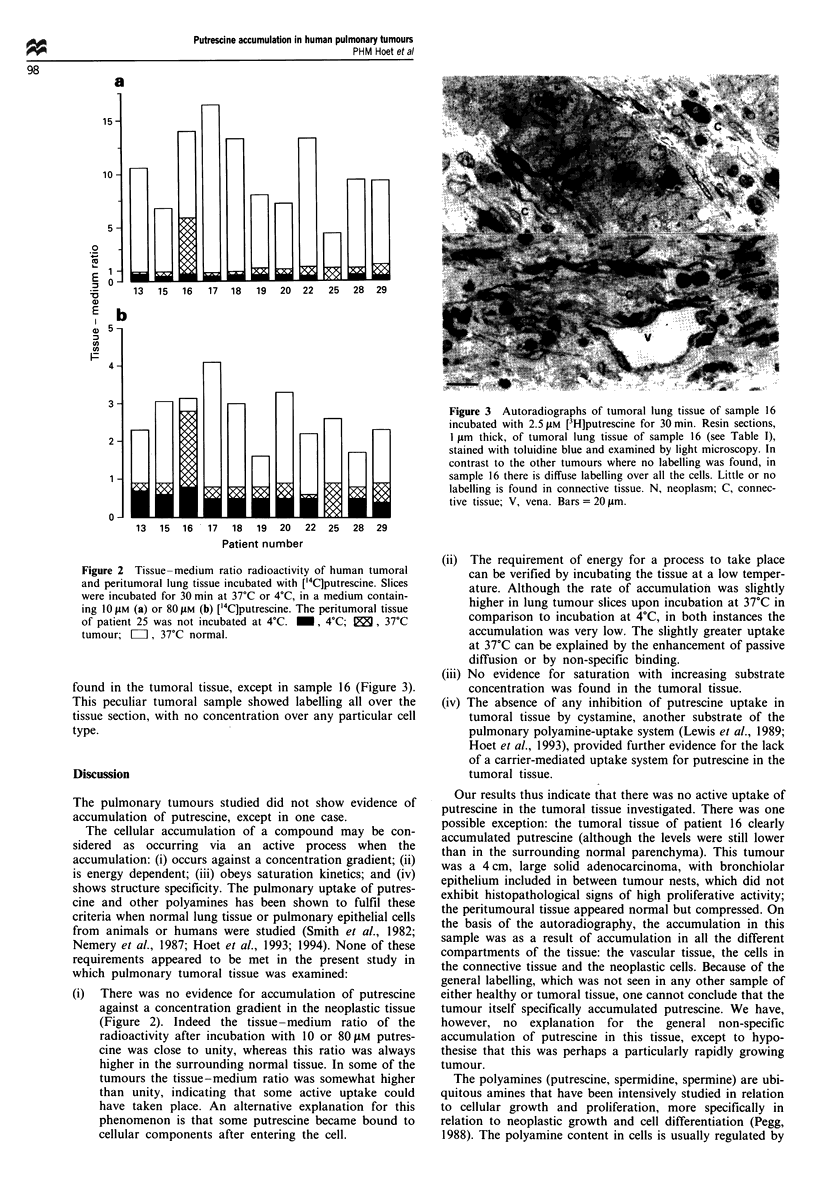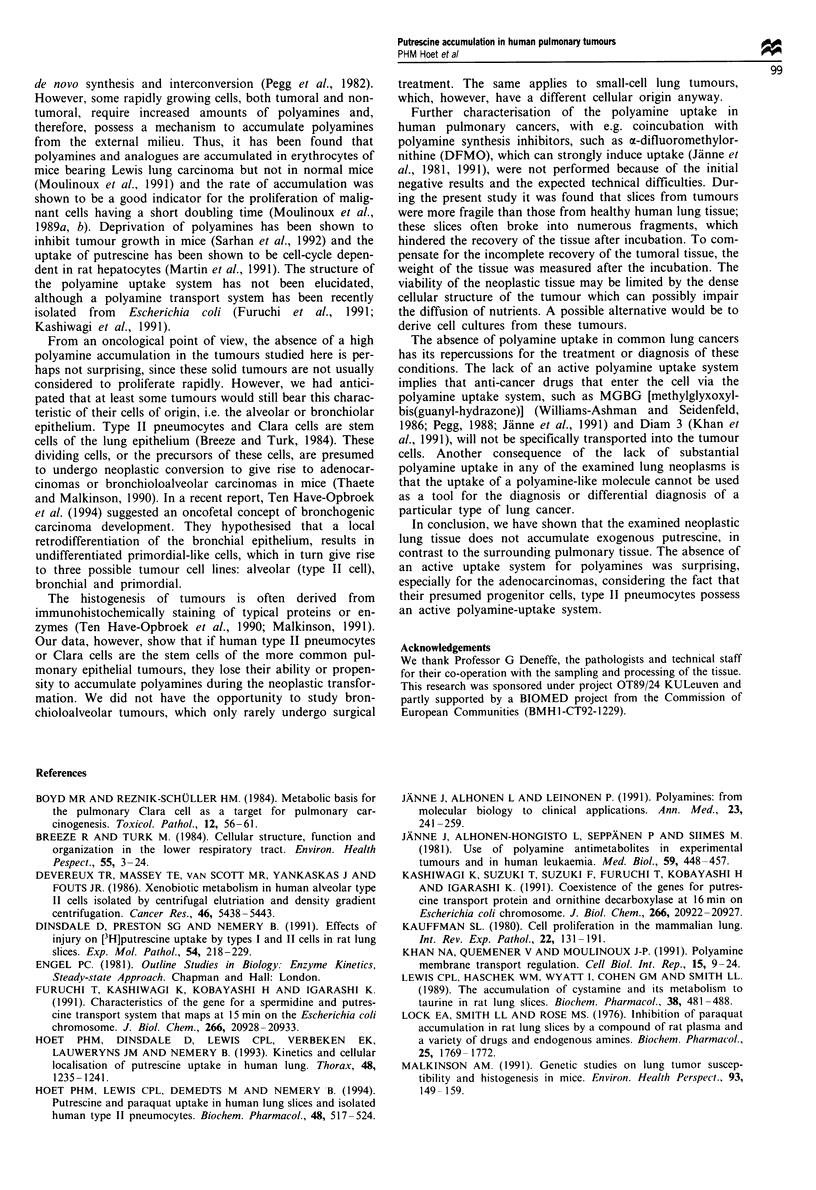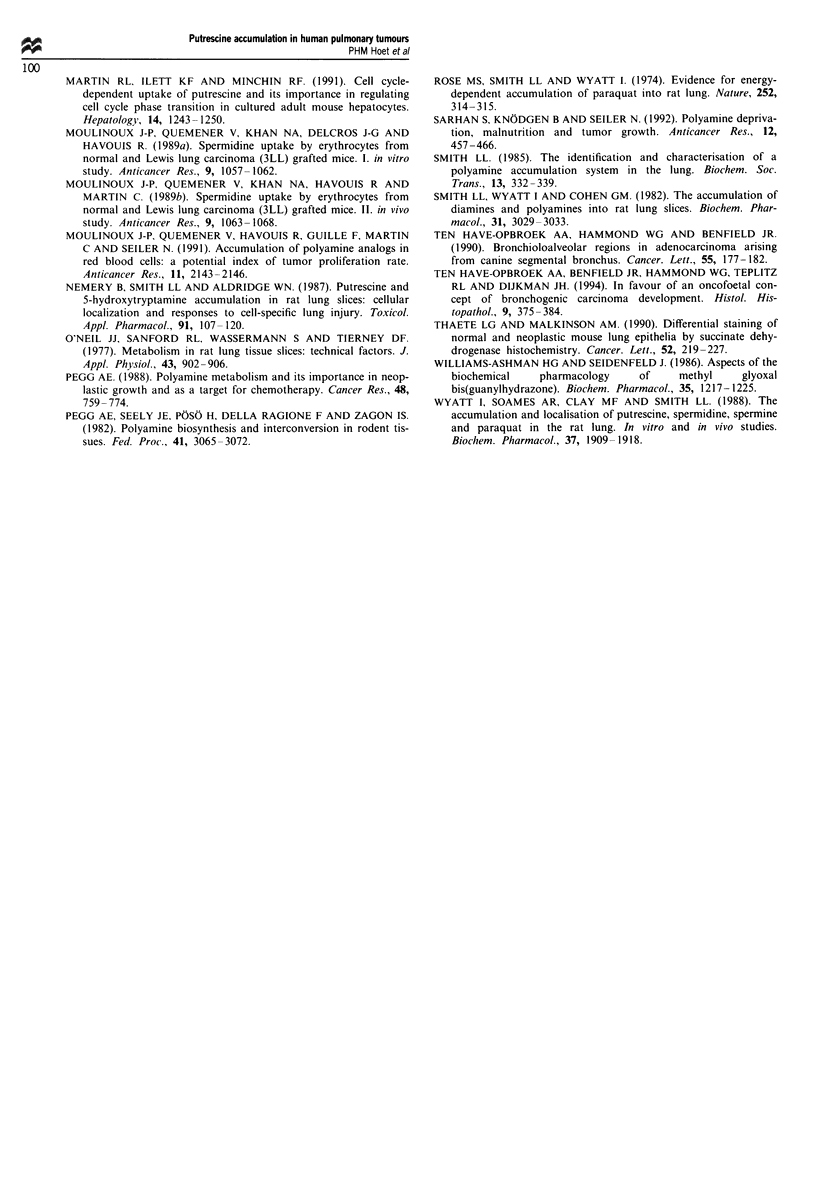# Putrescine accumulation in human pulmonary tumours.

**DOI:** 10.1038/bjc.1996.17

**Published:** 1996-01

**Authors:** P. H. Hoet, D. Dinsdale, E. K. Verbeken, M. Demedts, B. Nemery

**Affiliations:** K.U. Leuven, Laboratorium voor Pneumologie, Belgium.

## Abstract

**Images:**


British Journal of Cancer (1996) 73, 96–100
© 1996 Stockton Press All rights reserved 0007–0920/96 $12.00

### Putrescine accumulation in human pulmonary tumours

PHM Hoet[1], D Dinsdale[2], EK Verbeken[3], M Demedts[1] and B Nemery[1]

[1]*K.U. Leuven, Laboratorium voor Pneumologie, Herestraat 49, B-3000 Leuven, Belgium;* [2]*Medical Research Council, Toxicology Unit, Hodgkin Building, University of Leicester, PO Box 138 Lancasterroad, Leicester LE1 9HN, UK;* [3]*K.U. Leuven, Departement voor Pathologie, Kapucijnenvoer 35, B-3000 Leuven, Belgium.*

**Summary** Type II pneumocytes and Clara cells, both epithelial cells that possess an active uptake system for polyamines, have been identified as possible precursor cells of at least some types of lung tumours. In this study we have investigated whether human pulmonary tumours exhibit putrescine uptake. Lung slices from both tumoral tissue and non-tumoral tissue, obtained from patients undergoing surgery for lung cancer, were incubated with radiolabelled putrescine at both 37°C and 4°C. The accumulation of putrescine was evaluated by its apparent kinetic parameters, in the presence or absence of cystamine, and by autoradiography. The investigated tumoral tissue (six squamous carcinomas and five adenocarcinomas) did not show accumulation of putrescine above that attributable to simple diffusion, except for one adenocarcinoma. In this specimen autoradiography showed that the accumulation was not specifically associated with any particular cell type, but that practically every cell accumulated putrescine. We conclude that human pulmonary tumours do not accumulate polyamines in a manner similar to normal pulmonary epithelial cells.

**Keywords:** polyamines; putrescine; human lung neoplasm; pulmonary epithelium

Much more than any other major organ examined, normal lung tissue appears to accumulate polyamines via an active uptake system obeying Michaelis–Menten kinetics (Smith *et al*., 1982). The propensity of normal lung tissue to accumulate polyamines was discovered after it had been found that the highly selective pneumotoxic herbicide paraquat is actively accumulated in lung tissue (Rose *et al*., 1974; Lock *et al*., 1976) and that this accumulation occurs because paraquat is 'mistaken' for the structurally somewhat similar polyamines (Smith, 1985). The site of this uptake has been convincingly demonstrated to be essentially made up by the alveolar epithelium, i.e. type I and type II pneumocytes, as shown by autoradiography in both animal and human lungs (Nemery *et al*., 1987; Wyatt *et al*., 1988; Dinsdale *et al*., 1991; Hoet *et al*., 1993). In addition, the non-ciliated bronchiolar (Clara) cells of the rat are also a site of uptake, at least *in vitro* (Nemery *et al*., 1987; Wyatt *et al*., 1988), but this could neither be confirmed or denied for the human lung (Hoet *et al*., 1993). Neither the alveolar cells nor the Clara cells are characterised by a high proliferation rate in the normal lung (Kauffman, 1980), and thus the reasons for the substantial accumulation of polyamines by lung tissue are still unclear.

Given that a high polyamine uptake appears to be a specific feature of lung epithelial cells, it was logical to investigate whether some pulmonary epithelium-derived tumours would also exhibit a high polyamine uptake, irrespective of their proliferation rate.

Despite intensive study, the histogenetic origin of pulmonary tumours is still vague for most of the tumour types. On the basis of findings obtained in mice (Thaete and Malkinson, 1990; Malkinson, 1991) and in dog (Ten Have-Opbroek *et al*., 1990), adenocarcinomas have been considered to derive from type II pneumocytes. The origin of squamous cell carcinomas is still unclear, but there are suggestions that both Clara cells and type II pneumocytes could be implicated in human pulmonary epithelial tumours (Boyd and Reznik-Schüller, 1984; Devereux *et al*., 1986). Thus, if some tumours derive from Clara cells or type II pneumocytes, it is conceivable that these tumours also take up putrescine actively, in a way similar to the normal Clara cells or type II pneumocytes.

Correspondence: B. Nemery
Received 17 February 1995; revised 10 July 1995; accepted 19 July 1995

In this study, human lung tumours have been incubated in the presence of radiolabelled putrescine, and we have compared the uptake of putrescine in the neoplastic tissue with that in the surrounding parenchyma.

### Materials and methods

### *Reagents*

[1,4-$^{14}$C]Putrescine dihydrochloride (110 mCi mmol$^{-1}$) and [1,4*n*-$^{3}$H]putrescine dihydrochloride (24 mCi mmol$^{-1}$) were purchased from Amersham International (Brussels, Belgium). Soluene 350 tissue solubiliser, Emulsifier Safe and Ultima Gold scintillants as well as plastic scintillation vials (20 ml and 5 ml) were purchased from Packard (Zellik, Belgium). Ilford Nuclear Research Emulsion Gel K2 and Ilford Phenisol were purchased from Ilford Photo (Brussels, Belgium). Putrescine dihydrochloride, glutaraldehyde and glucose were purchased from Sigma Germany (Filterservice, Eupen, Belgium). All other chemicals were obtained from U.C.B. Belgium (Vel, Leuven, Belgium).

### *Preparation and incubation of lung slices*

Both normal and tumoral lung tissue samples were obtained from 11 patients (one female, aged 64 years; ten males, aged 57–77 years) undergoing lobectomy or pneumonectomy for lung cancer (Table I). (Data from the normal tissue of ten of these subjects are included in a previously published article; Hoet *et al*., 1993). Approval for using this type of tissue was obtained from the Ethical Committee of the Faculty of Medicine. Within minutes after resection, large portions of normal and tumoral tissue were dissected from the surgical specimen. The tissue pieces were placed in separate beakers in Krebs Ringer phosphate buffer (KRPB), containing sodium chloride (130 mM), potassium chloride (5.4 mM), calcium chloride (1.9 mM), magnesium sulphate (1.29 mM), disodium hydrogen phosphate (10 mM), glucose (11 mM) (pH 7.4) at room temperature.

Within 1 h, 0.7 mm-thick slices were prepared from both normal and tumoral tissues with a McIlwain tissue chopper (Mickle Laboratories, Surrey, UK). The slices, with cut surfaces of about 0.5 cm$^2$, were weighed and incubated in batches of approximately 30 mg in 3 ml of KRPB in 40 ml polyethylene flasks placed in a shaking water bath (120–140

**Table I** Characteristics of patients ranked by sex and age

| *Patient no.* | *Sex* | *Age (years)* | *Final diagnosis* | *Parenchyma*[a] |
|---|---|---|---|---|
| 19 | F | 64 | Adenocarcinoma | Normal |
| 15 | M | 57 | Squamous carcinoma | Normal |
| 17 | M | 63 | Squamous carcinoma | Normal |
| 29 | M | 63 | Adenocarcinoma | Normal |
| 20 | M | 64 | Squamous carcinoma | Emphysema |
| 16 | M | 64 | Adenocarcinoma | Normal |
| 22 | M | 65 | Squamous carcinoma | Normal |
| 13 | M | 66 | Squamous carcinoma | Normal |
| 18 | M | 67 | Squamous carcinoma | Normal |
| 25 | M | 69 | Adenocarcinoma | Normal |
| 28 | M | 77 | Adenocarcinoma | Normal |

[a]Histological appearance of peritumoral parenchyma. F, female; M, male.

strokes min$^{-1}$) at 37°C (or at 4°C) (O'Neil *et al.*, 1977). The slices were incubated in KRPB containing putrescine (2.5–80 μM) and [1,4-$^{14}$C]putrescine (0.1 Ci per incubation flask) in the presence or absence of 50 μM cystamine. Putrescine uptake was determined as described previously (Hoet *et al.*, 1993), by measuring tissue-associated radioactivity.

The tissue–medium ratio of radioactivity was calculated as: (tissue radioactivity/0.7)/(medium radioactivity post incubation), with the factor 0.7 representing the 70% aqueous portion of the tissue containing free putrescine.

### *Autoradiography*

Lung slices were incubated for 30 min at 37°C in KRPB containing 2.5 μM putrescine and [1,4*n*-$^{3}$H]putrescine (500 μCi μmol$^{-1}$).

At the end of the incubation, the slices were fixed in 6.5% glutaraldehyde in 0.1% cacodylate buffer (pH 7.4) and prepared for autoradiography, as previously described by Nemery *et al.* (1987) and Dinsdale *et al.* (1991). Slices for light microscopy were embedded in EPON 812 resin and sections of 1 μm thickness were mounted onto microscope slides. The slides were dipped at 40°C into an Ilford K2 nuclear emulsion (25%), dried, and kept at 4°C in the dark. After 50 days, the slides were developed (Ilford Phenisol, 15 min), fixed (1% acetic acid 2 min, 30% sodium thiosulphate) and stained (0.1% toluidine blue in 0.1% borax, 37°C, 9 min).

### *Analysis of data*

When intra-individual duplicate or triplicate determinations were available, an average value was taken. The apparent accumulation obtained at 37°C was considered to represent the sum of the active uptake and the passive uptake by diffusion, with the latter being estimated from the incubations at 4°C. All weights are wet weights.

The maximum rate of uptake, $V_{max}$ (expressed in nmol g$^{-1}$ h$^{-1}$), is achieved at infinite substrate concentration, and $K_m$ (expressed in μM substrate) is the medium concentration at which the rate of uptake is half $V_{max}$. The apparent kinetic parameters were calculated from a Hanes–Woolf plot (Engel, 1981).

Data from control and treated slices were compared by Student's *t*-test for paired data, or by analysis of variance with Duncan grouping, using the SAS/STAT package (6th version). The level of significance was set at $P<0.05$.

### Results

### *Putrescine accumulation*

The macroscopically normal tissue accumulated putrescine, in an active manner, as described previously (Hoet *et al.*, 1993) (Figure 1). In the present group of subjects, this uptake was characterised by a mean $K_m$ of 9.9 μM and a mean $V_{max}$ of 297 nmol g$^{-1}$ h$^{-1}$. No differences were found between tissue from patients with adenocarcinoma ($K_m$ 10.2 μM, $V_{max}$ 384 nmol g$^{-1}$ h$^{-1}$) or squamous carcinoma ($K_m$ 10.2 μM, $V_{max}$ 218 nmol g$^{-1}$ h$^{-1}$).

**Figure 1** Accumulation of [$^{14}$C]putrescine in normal and tumoral tissue. Slices from human lung prepared from normal parenchyma and from the tumour were incubated for 30 min at 37°C or 4°C, in medium containing various concentrations of [$^{14}$C]putrescine (2.5–80 μM). Values obtained for the accumulation of putrescine in normal lung slices at 4°C (not shown) were not subtracted from those obtained at 37°C (○). The values for tumour lung slices were split between adenocarcinomas ($n = 5$) (▲) and squamous carcinomas ($n = 6$) (▼), the values obtained at 4°C (■) were not subtracted.

In contrast, the accumulation of putrescine in tumoral tissue was linear with the medium concentration, both at 4°C and 37°C. When the data were grouped according to type of neoplasm, no differences in the rate of putrescine accumulation were found.

Figure 2 shows that, in the tumoral tissue, the tissue–medium ratio of radioactivity was usually not higher than 1, indicating that the measured tissue radioactivity was essentially due to passive diffusion into the tissue. In one sample (sample 16) a higher ratio was observed.

Co-incubation of normal parenchyma with putrescine in the presence of 50 μM cystamine, a known competitive inhibitor of putrescine uptake (Lewis *et al.*, 1989), resulted, as previously reported (Hoet *et al.*, 1993), in reduced accumulation of label (data not shown). In contrast, uptake by tumoral tissue was not affected by cystamine, except in samples from patient 16. In this sample the uptake was reduced, as in non-tumoral tissue, but levels were still higher than those attributable to passive diffusion.

### *Autoradiography*

Autoradiography was consistent with the data obtained for the accumulation of [1,4-$^{14}$C]putrescine: no labelling was

**Figure 2** Tissue–medium ratio radioactivity of human tumoral and peritumoral lung tissue incubated with [$^{14}$C]putrescine. Slices were incubated for 30 min at 37°C or 4°C, in a medium containing 10 μM (**a**) or 80 μM (**b**) [$^{14}$C]putrescine. The peritumoral tissue of patient 25 was not incubated at 4°C. ■, 4°C; ▨, 37°C tumour; □, 37°C normal.

**Figure 3** Autoradiographs of tumoral lung tissue of sample 16 incubated with 2.5 μM [$^3$H]putrescine for 30 min. Resin sections, 1 μm thick, of tumoral lung tissue of sample 16 (see Table I), stained with toluidine blue and examined by light microscopy. In contrast to the other tumours where no labelling was found, in sample 16 there is diffuse labelling over all the cells. Little or no labelling is found in connective tissue. N, neoplasm; C, connective tissue; V, vena. Bars = 20 μm.

found in the tumoral tissue, except in sample 16 (Figure 3). This peculiar tumoral sample showed labelling all over the tissue section, with no concentration over any particular cell type.

### Discussion

The pulmonary tumours studied did not show evidence of accumulation of putrescine, except in one case.

The cellular accumulation of a compound may be considered as occurring via an active process when the accumulation: (i) occurs against a concentration gradient; (ii) is energy dependent; (iii) obeys saturation kinetics; and (iv) shows structure specificity. The pulmonary uptake of putrescine and other polyamines has been shown to fulfil these criteria when normal lung tissue or pulmonary epithelial cells from animals or humans were studied (Smith *et al.*, 1982; Nemery *et al.*, 1987; Hoet *et al.*, 1993; 1994). None of these requirements appeared to be met in the present study in which pulmonary tumoral tissue was examined:

(i) There was no evidence for accumulation of putrescine against a concentration gradient in the neoplastic tissue (Figure 2). Indeed the tissue–medium ratio of the radioactivity after incubation with 10 or 80 μM putrescine was close to unity, whereas this ratio was always higher in the surrounding normal tissue. In some of the tumours the tissue–medium ratio was somewhat higher than unity, indicating that some active uptake could have taken place. An alternative explanation for this phenomenon is that some putrescine became bound to cellular components after entering the cell.

(ii) The requirement of energy for a process to take place can be verified by incubating the tissue at a low temperature. Although the rate of accumulation was slightly higher in lung tumour slices upon incubation at 37°C in comparison to incubation at 4°C, in both instances the accumulation was very low. The slightly greater uptake at 37°C can be explained by the enhancement of passive diffusion or by non-specific binding.

(iii) No evidence for saturation with increasing substrate concentration was found in the tumoral tissue.

(iv) The absence of any inhibition of putrescine uptake in tumoral tissue by cystamine, another substrate of the pulmonary polyamine-uptake system (Lewis *et al.*, 1989; Hoet *et al.*, 1993), provided further evidence for the lack of a carrier-mediated uptake system for putrescine in the tumoral tissue.

Our results thus indicate that there was no active uptake of putrescine in the tumoral tissue investigated. There was one possible exception: the tumoral tissue of patient 16 clearly accumulated putrescine (although the levels were still lower than in the surrounding normal parenchyma). This tumour was a 4 cm, large solid adenocarcinoma, with bronchiolar epithelium included in between tumour nests, which did not exhibit histopathological signs of high proliferative activity; the peritumoural tissue appeared normal but compressed. On the basis of the autoradiography, the accumulation in this sample was as a result of accumulation in all the different compartments of the tissue: the vascular tissue, the cells in the connective tissue and the neoplastic cells. Because of the general labelling, which was not seen in any other sample of either healthy or tumoral tissue, one cannot conclude that the tumour itself specifically accumulated putrescine. We have, however, no explanation for the general non-specific accumulation of putrescine in this tissue, except to hypothesise that this was perhaps a particularly rapidly growing tumour.

The polyamines (putrescine, spermidine, spermine) are ubiquitous amines that have been intensively studied in relation to cellular growth and proliferation, more specifically in relation to neoplastic growth and cell differentiation (Pegg, 1988). The polyamine content in cells is usually regulated by

*de novo* synthesis and interconversion (Pegg *et al*., 1982). However, some rapidly growing cells, both tumoral and non-tumoral, require increased amounts of polyamines and, therefore, possess a mechanism to accumulate polyamines from the external milieu. Thus, it has been found that polyamines and analogues are accumulated in erythrocytes of mice bearing Lewis lung carcinoma but not in normal mice (Moulinoux *et al*., 1991) and the rate of accumulation was shown to be a good indicator for the proliferation of malignant cells having a short doubling time (Moulinoux *et al*., 1989*a*, *b*). Deprivation of polyamines has been shown to inhibit tumour growth in mice (Sarhan *et al*., 1992) and the uptake of putrescine has been shown to be cell-cycle dependent in rat hepatocytes (Martin *et al*., 1991). The structure of the polyamine uptake system has not been elucidated, although a polyamine transport system has been recently isolated from *Escherichia coli* (Furuchi *et al*., 1991; Kashiwagi *et al*., 1991).

From an oncological point of view, the absence of a high polyamine accumulation in the tumours studied here is perhaps not surprising, since these solid tumours are not usually considered to proliferate rapidly. However, we had anticipated that at least some tumours would still bear this characteristic of their cells of origin, i.e. the alveolar or bronchiolar epithelium. Type II pneumocytes and Clara cells are stem cells of the lung epithelium (Breeze and Turk, 1984). These dividing cells, or the precursors of these cells, are presumed to undergo neoplastic conversion to give rise to adenocarcinomas or bronchioloalveolar carcinomas in mice (Thaete and Malkinson, 1990). In a recent report, Ten Have-Opbroek *et al*. (1994) suggested an oncofetal concept of bronchogenic carcinoma development. They hypothesised that a local retrodifferentiation of the bronchial epithelium, results in undifferentiated primordial-like cells, which in turn give rise to three possible tumour cell lines: alveolar (type II cell), bronchial and primordial.

The histogenesis of tumours is often derived from immunohistochemically staining of typical proteins or enzymes (Ten Have-Opbroek *et al*., 1990; Malkinson, 1991). Our data, however, show that if human type II pneumocytes or Clara cells are the stem cells of the more common pulmonary epithelial tumours, they lose their ability or propensity to accumulate polyamines during the neoplastic transformation. We did not have the opportunity to study bronchioloalveolar tumours, which only rarely undergo surgical treatment. The same applies to small-cell lung tumours, which, however, have a different cellular origin anyway.

Further characterisation of the polyamine uptake in human pulmonary cancers, with e.g. coincubation with polyamine synthesis inhibitors, such as α-difluoromethylornithine (DFMO), which can strongly induce uptake (Jänne *et al*., 1981, 1991), were not performed because of the initial negative results and the expected technical difficulties. During the present study it was found that slices from tumours were more fragile than those from healthy human lung tissue; these slices often broke into numerous fragments, which hindered the recovery of the tissue after incubation. To compensate for the incomplete recovery of the tumoral tissue, the weight of the tissue was measured after the incubation. The viability of the neoplastic tissue may be limited by the dense cellular structure of the tumour which can possibly impair the diffusion of nutrients. A possible alternative would be to derive cell cultures from these tumours.

The absence of polyamine uptake in common lung cancers has its repercussions for the treatment or diagnosis of these conditions. The lack of an active polyamine uptake system implies that anti-cancer drugs that enter the cell via the polyamine uptake system, such as MGBG [methylglyxoxyl-bis(guanyl-hydrazone)] (Williams-Ashman and Seidenfeld, 1986; Pegg, 1988; Jänne *et al*., 1991) and Diam 3 (Khan *et al*., 1991), will not be specifically transported into the tumour cells. Another consequence of the lack of substantial polyamine uptake in any of the examined lung neoplasms is that the uptake of a polyamine-like molecule cannot be used as a tool for the diagnosis or differential diagnosis of a particular type of lung cancer.

In conclusion, we have shown that the examined neoplastic lung tissue does not accumulate exogenous putrescine, in contrast to the surrounding pulmonary tissue. The absence of an active uptake system for polyamines was surprising, especially for the adenocarcinomas, considering the fact that their presumed progenitor cells, type II pneumocytes possess an active polyamine-uptake system.

**Acknowledgements**

We thank Professor G Deneffe, the pathologists and technical staff for their co-operation with the sampling and processing of the tissue. This research was sponsored under project OT89/24 KULeuven and partly supported by a BIOMED project from the Commission of European Communities (BMH1-CT92-1229).

### References

BOYD MR AND REZNIK-SCHÜLLER HM. (1984). Metabolic basis for the pulmonary Clara cell as a target for pulmonary carcinogenesis. *Toxicol. Pathol*., **12,** 56–61.

BREEZE R AND TURK M. (1984). Cellular structure, function and organization in the lower respiratory tract. *Environ. Health Pespect*., **55,** 3–24.

DEVEREUX TR, MASSEY TE, VAN SCOTT MR, YANKASKAS J AND FOUTS JR. (1986). Xenobiotic metabolism in human alveolar type II cells isolated by centrifugal elutriation and density gradient centrifugation. *Cancer Res*., **46,** 5438–5443.

DINSDALE D, PRESTON SG AND NEMERY B. (1991). Effects of injury on [$^3$H]putrescine uptake by types I and II cells in rat lung slices. *Exp. Mol. Pathol*., **54,** 218–229.

ENGEL PC. (1981). *Outline Studies in Biology: Enzyme Kinetics, Steady-state Approach*. Chapman and Hall: London.

FURUCHI T, KASHIWAGI K, KOBAYASHI H AND IGARASHI K. (1991). Characteristics of the gene for a spermidine and putrescine transport system that maps at 15 min on the *Escherichia coli* chromosome. *J. Biol. Chem*., **266,** 20928–20933.

HOET PHM, DINSDALE D, LEWIS CPL, VERBEKEN EK, LAUWERYNS JM AND NEMERY B. (1993). Kinetics and cellular localisation of putrescine uptake in human lung. *Thorax*, **48,** 1235–1241.

HOET PHM, LEWIS CPL, DEMEDTS M AND NEMERY B. (1994). Putrescine and paraquat uptake in human lung slices and isolated human type II pneumocytes. *Biochem. Pharmacol*., **48,** 517–524.

JÄNNE J, ALHONEN L AND LEINONEN P. (1991). Polyamines: from molecular biology to clinical applications. *Ann. Med*., **23,** 241–259.

JÄNNE J, ALHONEN-HONGISTO L, SEPPÄNEN P AND SIIMES M. (1981). Use of polyamine antimetabolites in experimental tumours and in human leukaemia. *Med. Biol*., **59,** 448–457.

KASHIWAGI K, SUZUKI T, SUZUKI F, FURUCHI T, KOBAYASHI H AND IGARASHI K. (1991). Coexistence of the genes for putrescine transport protein and ornithine decarboxylase at 16 min on *Escherichia coli* chromosome. *J. Biol. Chem*., **266,** 20922–20927.

KAUFFMAN SL. (1980). Cell proliferation in the mammalian lung. *Int. Rev. Exp. Pathol*., **22,** 131–191.

KHAN NA, QUEMENER V AND MOULINOUX J-P. (1991). Polyamine membrane transport regulation. *Cell Biol. Int. Rep*., **15,** 9–24.

LEWIS CPL, HASCHEK WM, WYATT I, COHEN GM AND SMITH LL. (1989). The accumulation of cystamine and its metabolism to taurine in rat lung slices. *Biochem. Pharmacol*., **38,** 481–488.

LOCK EA, SMITH LL AND ROSE MS. (1976). Inhibition of paraquat accumulation in rat lung slices by a compound of rat plasma and a variety of drugs and endogenous amines. *Biochem. Pharmacol*., **25,** 1769–1772.

MALKINSON AM. (1991). Genetic studies on lung tumor susceptibility and histogenesis in mice. *Environ. Health Perspect*., **93,** 149–159.

MARTIN RL, ILETT KF AND MINCHIN RF. (1991). Cell cycle-dependent uptake of putrescine and its importance in regulating cell cycle phase transition in cultured adult mouse hepatocytes. *Hepatology*, **14**, 1243–1250.

MOULINOUX J-P, QUEMENER V, KHAN NA, DELCROS J-G AND HAVOUIS R. (1989*a*). Spermidine uptake by erythrocytes from normal and Lewis lung carcinoma (3LL) grafted mice. I. *in vitro* study. *Anticancer Res.*, **9**, 1057–1062.

MOULINOUX J-P, QUEMENER V, KHAN NA, HAVOUIS R AND MARTIN C. (1989*b*). Spermidine uptake by erythrocytes from normal and Lewis lung carcinoma (3LL) grafted mice. II. *in vivo* study. *Anticancer Res.*, **9**, 1063–1068.

MOULINOUX J-P, QUEMENER V, HAVOUIS R, GUILLE F, MARTIN C AND SEILER N. (1991). Accumulation of polyamine analogs in red blood cells: a potential index of tumor proliferation rate. *Anticancer Res.*, **11**, 2143–2146.

NEMERY B, SMITH LL AND ALDRIDGE WN. (1987). Putrescine and 5-hydroxytryptamine accumulation in rat lung slices: cellular localization and responses to cell-specific lung injury. *Toxicol. Appl. Pharmacol.*, **91**, 107–120.

O'NEIL JJ, SANFORD RL, WASSERMANN S AND TIERNEY DF. (1977). Metabolism in rat lung tissue slices: technical factors. *J. Appl. Physiol.*, **43**, 902–906.

PEGG AE. (1988). Polyamine metabolism and its importance in neoplastic growth and as a target for chemotherapy. *Cancer Res.*, **48**, 759–774.

PEGG AE, SEELY JE, PÖSÖ H, DELLA RAGIONE F AND ZAGON IS. (1982). Polyamine biosynthesis and interconversion in rodent tissues. *Fed. Proc.*, **41**, 3065–3072.

ROSE MS, SMITH LL AND WYATT I. (1974). Evidence for energy-dependent accumulation of paraquat into rat lung. *Nature*, **252**, 314–315.

SARHAN S, KNÖDGEN B AND SEILER N. (1992). Polyamine deprivation, malnutrition and tumor growth. *Anticancer Res.*, **12**, 457–466.

SMITH LL. (1985). The identification and characterisation of a polyamine accumulation system in the lung. *Biochem. Soc. Trans.*, **13**, 332–339.

SMITH LL, WYATT I AND COHEN GM. (1982). The accumulation of diamines and polyamines into rat lung slices. *Biochem. Pharmacol.*, **31**, 3029–3033.

TEN HAVE-OPBROEK AA, HAMMOND WG AND BENFIELD JR. (1990). Bronchioloalveolar regions in adenocarcinoma arising from canine segmental bronchus. *Cancer. Lett.*, **55**, 177–182.

TEN HAVE-OPBROEK AA, BENFIELD JR, HAMMOND WG, TEPLITZ RL AND DIJKMAN JH. (1994). In favour of an oncofoetal concept of bronchogenic carcinoma development. *Histol. Histopathol.*, **9**, 375–384.

THAETE LG AND MALKINSON AM. (1990). Differential staining of normal and neoplastic mouse lung epithelia by succinate dehydrogenase histochemistry. *Cancer. Lett.*, **52**, 219–227.

WILLIAMS-ASHMAN HG AND SEIDENFELD J. (1986). Aspects of the biochemical pharmacology of methyl glyoxal bis(guanylhydrazone). *Biochem. Pharmacol.*, **35**, 1217–1225.

WYATT I, SOAMES AR, CLAY MF AND SMITH LL. (1988). The accumulation and localisation of putrescine, spermidine, spermine and paraquat in the rat lung. *In vitro* and *in vivo* studies. *Biochem. Pharmacol.*, **37**, 1909–1918.